# Cortical thickness, surface area, and folding alterations in male youths with conduct disorder and varying levels of callous–unemotional traits

**DOI:** 10.1016/j.nicl.2015.04.018

**Published:** 2015-04-30

**Authors:** Graeme Fairchild, Nicola Toschi, Cindy C. Hagan, Ian M. Goodyer, Andrew J. Calder, Luca Passamonti

**Affiliations:** a Academic Unit of Psychology, University of Southampton, Southampton, UK; b Department of Psychiatry, University of Cambridge, Cambridge, UK; c Department of Biomedicine and Prevention, University of Rome, Rome , Italy; d Department of Radiology, Athinoula A. Martinos Center for Biomedical Imaging, Boston, USA; e Harvard Medical School, Boston, MA, USA; f Department of Psychology, Columbia University, New York, NY, USA; g Medical Research Council Cognition and Brain Sciences Unit, Cambridge, UK; h Institute of Bioimaging and Molecular Physiology, National Research Council, Catanzaro, Italy; i Department of Clinical Neurosciences, University of Cambridge, Cambridge, UK

**Keywords:** Surface based morphometry, Conduct disorder, Developmental taxonomic theory, Callous–unemotional traits

## Abstract

**Purpose:**

Previous studies have reported changes in gray matter volume in youths with conduct disorder (CD), although these differences are difficult to interpret as they may have been driven by alterations in cortical thickness, surface area (SA), or folding. The objective of this study was to use surface-based morphometry (SBM) methods to compare male youths with CD and age and sex-matched healthy controls (HCs) in cortical thickness, SA, and folding. We also tested for structural differences between the childhood-onset and adolescence-onset subtypes of CD and performed regression analyses to assess for relationships between CD symptoms and callous–unemotional (CU) traits and SBM-derived measures.

**Methods:**

We acquired structural neuroimaging data from 20 HCs and 36 CD participants (18 with childhood-onset CD and 18 with adolescence-onset CD) and analyzed the data using FreeSurfer.

**Results:**

Relative to HCs, youths with CD showed reduced cortical thickness in the superior temporal gyrus, reduced SA in the orbitofrontal cortex (OFC), and increased cortical folding in the insula. There were no significant differences between the childhood-onset and adolescence-onset CD subgroups in cortical thickness or SA, but several frontal and temporal regions showed increased cortical folding in childhood-onset relative to adolescence-onset CD participants. Both CD subgroups also showed increased cortical folding relative to HCs. CD symptoms were negatively correlated with OFC SA whereas CU traits were positively correlated with insula folding.

**Conclusions:**

Cortical thinning in the superior temporal gyrus may contribute to the social cognitive impairments displayed by youths with CD, whereas reduced OFC SA may lead to impairments in emotion regulation and reward processing in youths with CD. The increased cortical folding observed in the insula may reflect a maturational delay in this region and could mediate the link between CU traits and empathy deficits. Altered cortical folding was observed in childhood-onset and adolescence-onset forms of CD.

## Highlights

• Surface based morphometry was used to study cortical structure in conduct disorder.
• Youths with CD showed superior temporal gyrus cortical thinning relative to controls.
• Increased insula cortical folding and reduced orbitofrontal cortex (OFC) surface area in CD.
• OFC surface area was negatively correlated with number of CD symptoms.
• Insula cortical folding was positively correlated with callous–unemotional traits.

## Graphical abstract

**Figure:**
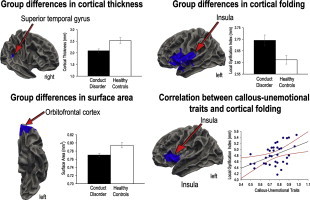

## 1 Introduction

Conduct disorder (CD) is a psychiatric condition that emerges in childhood or adolescence and is characterized by a pervasive pattern of antisocial behavior (American Psychiatric Association, 2013). Previous structural imaging studies using voxel-based morphometry (VBM) have reported reduced gray matter volume in the orbitofrontal cortex, dorsomedial prefrontal cortex, anterior insular cortex, fusiform gyrus and occipital cortex in youths with CD relative to healthy controls (HCs) (Fairchild et al., 2011; Sterzer et al., 2007; Fahim et al., 2011; Huebner et al., 2008). However, the volumetric differences between CD and HC participants that were identified in previous VBM studies may have been driven by changes in cortical thickness, surface area (SA), or local gyrification index (lGI), a measure of cortical folding, or by a combination of these measures (Hutton et al., 2009).

Surface-based morphometry (SBM) methods enable researchers to disaggregate these interrelated measures and examine how each of these metrics contributes to changes in brain anatomy. This is important because cortical thickness, SA, and lGI have distinct developmental trajectories and reflect different cellular mechanisms (Raznahan et al., 2011; Rakic, 2009). Specifically, cortical thickness is determined by the horizontal layers in the cortical columns including neurons and neuropil, whereas SA reflects the number of radial columns perpendicular to the pial surface (Rakic, 2009). Conversely, lGI refers to the folding patterns at the brain's surface and relates to the microstructure of the neuronal sheets (Zilles et al., 1989). It has also been suggested that local axonal connectivity within a cortical region determines its degree of folding (Zilles et al., 1989). Furthermore, cortical thickness, SA and lGI display different developmental trajectories, with cortical thickness and SA peaking at ages 8.5 and 9 years, respectively, whereas lGI peaks at around age 1.5 years (Raznahan et al., 2011). Finally, there is some evidence that SBM methods are more sensitive in detecting gray matter alterations than VBM (Hutton et al., 2009), although SBM methods are not informative about subcortical changes.

Relative to research using VBM, few studies have employed SBM methods to investigate brain structure in CD. The first SBM study in this field observed reduced cortical thickness in the superior temporal gyrus (STG), insula, and orbitofrontal cortex (OFC) in children with Oppositional Defiant Disorder (ODD) or CD, relative to HCs (Fahim et al., 2011). Another study found reduced STG thickness and folding deficits in the insula and OFC in CD adolescents without comorbid Attention-Deficit/Hyperactivity Disorder (ADHD), compared with HCs (Hyatt et al., 2012). Very recently, Wallace et al. (2014) found that CD adolescents with callous–unemotional (CU) traits, a personality factor reflecting emotional detachment and deficits in empathy (Frick and White, 2008), showed reduced cortical thickness in the STG and inferior parietal cortex, relative to HCs (Wallace et al., 2014). The same authors also observed a negative correlation between CU traits and STG thickness, but no group differences or significant correlations for lGI or SA (Wallace et al., 2014). Overall, these studies have provided important insights regarding the relationship between brain structure and CD, but were subject to certain limitations that made it difficult to interpret the findings. Specifically, one of the earlier studies (Fahim et al., 2011) recruited children with ODD and CD diagnoses, which is problematic as these disorders may have different etiologies. In addition, all of the previous studies included male and female participants and, with the exception of the study by Wallace et al. (2014), did not control for intelligence quotient (IQ), socioeconomic status (SES), or ADHD comorbidity. These recruitment strategies may be problematic for a number of reasons.

First, there is evidence for sex differences in brain structure and sexually-dimorphic trajectories of brain development in typically-developing youths, as well as those with psychiatric disorders (Raznahan et al., 2011; Fairchild et al., 2013a). Hence, collapsing across males and females without having sufficiently large sample sizes may lead to incorrect conclusions or obscure group differences if the relationship between CD and brain structure differs between the sexes, as suggested by our recent VBM study of males and females with CD (Fairchild et al., 2013a). Furthermore, the developmental course of antisocial behavior may differ between males and females (Fontaine et al., 2009). Finally, the relationship between SBM measures and CU traits in mixed samples of males and females may be confounded by gender, because CU traits tend to be higher in males than females (Pechorro et al., 2013). To address this issue, our study was restricted to males alone.

Second, the majority of the previous SBM studies in this area did not control for IQ or SES, two factors that have been consistently associated with CD (Murray and Farrington, 2010). Previous studies have shown that IQ and SES are both related to cortical thickness and lGI (Raznahan et al., 2011; Lawson et al., 2013); hence, it is important to match CD and HC groups in terms of IQ and SES, to ensure that group differences in cortical structure are not explained by group differences in cognitive ability or socio-demographic characteristics. Consequently, we deliberately matched the CD and HC groups on these key variables by selecting individuals from a larger sample.

Third, there is substantial overlap between CD and ADHD, with many children and adolescents with CD showing at least some symptoms of ADHD and a significant proportion fulfilling formal diagnostic criteria for ADHD (Klein et al., 1997). Consequently, it is important to investigate the contribution of ADHD comorbidity to the SBM differences observed in CD populations. Although still valuable, previous studies either excluded CD participants with comorbid ADHD or did not assess the effect of ADHD on the SBM findings. This means that the impact of ADHD comorbidity on changes in cortical thickness, surface area or folding in CD is not well understood, although the study by Wallace et al. showed that reductions in cortical thickness in the right superior temporal gyrus in CD remained significant when excluding participants with comorbid ADHD (Wallace et al., 2014). To examine the effects of ADHD comorbidity on the SBM results, we ran our analyses twice, first controlling for lifetime ADHD symptoms to examine which of the group effects were specifically related to CD and second without including ADHD symptoms as a covariate to investigate whether additional or distinct SBM findings were obtained when studying a CD sample that is more representative of clinical reality.

Lastly, earlier studies either did not assess the age-of-onset of CD (Wallace et al., 2014), which is considered an important distinction in the classification of CD (American Psychiatric Association, 2013), or included too few participants with each CD subtype to compare the childhood-onset (CO-CD) and adolescence-onset (AO-CD) variants of CD (Hyatt et al., 2012). To overcome these issues and investigate whether individuals with CO-CD and AO-CD differ from each other in SBM measures, the present study recruited male adolescents and young adults with either CO-CD or AO-CD and examined whether they show similar or distinct alterations in cortical thickness, surface area or folding relative to HCs. Given our previous work (Fairchild et al., 2011), we predicted that both subgroups would show alterations in SBM measures, although such differences would be most pronounced in the CO-CD group. Furthermore, we also investigated the impact of individual differences in CU traits and CD symptoms on SBM measures.

We hypothesized that youths with CD, relative to HCs, would show reduced cortical thickness, SA and lGI in regions previously implicated in social cognition, emotion regulation, and decision-making (i.e., the STG, insula, and OFC). Alterations in these regions were also predicted on the basis of earlier behavioral studies showing that adolescents with CD display deficits in decision-making, emotion recognition, and social cognition (Fairchild et al., 2009a,b). Consistent with our previous work using VBM methods (Fairchild et al., 2011, 2013a), we predicted that group differences in SBM metrics would be attenuated, but would remain significant, when controlling for ADHD symptoms in the statistical analyses. Finally, we hypothesized that CU traits and CD symptoms would be *negatively* correlated with cortical thickness, surface area, and lGI (Wallace et al., 2014).

## 2 Materials and methods

### 2.1 Participants

Thirty-six male participants with CD and 20 male HCs (aged 16–21 years) were selected from an original sample of 92 adolescents and young adults assessed in a series of structural and functional neuroimaging studies comparing CD and HC individuals (Passamonti et al., 2010; Fairchild et al., 2011). This larger dataset enabled us to deliberately match the CD and HC groups in terms of potentially confounding variables such as age, sex, IQ, and SES (Raznahan et al., 2011; Lawson et al., 2013).

All participants and their parents were assessed for CD and other common disorders using the Schedule for Affective Disorders and Schizophrenia for School-Age Children-Present and Lifetime Version (K-SADS-PL) (Kaufman et al., 1997). The interviews were performed in separate rooms and diagnoses were reached by combining information across both interviews. Participants with CD were classified as having CO-CD if they or their parents reported the presence of at least one CD symptom and functional impairment before age 10 (American Psychiatric Association, 2013). Alternatively, if no CD symptoms were reported by either informant before age 10 years but the participant subsequently developed CD, an AO-CD diagnosis was given. According to these criteria, 18 CD participants were classified as having CO-CD and 18 as having AO-CD. CU traits were assessed using the callous–unemotional dimension subscale of the Youth Psychopathic traits Inventory (YPI) (Andershed et al., 2002).

Participants with CD were recruited from schools and colleges, pupil referral units and the Cambridge Youth Offending Service, whereas HCs were recruited from schools and colleges. Exclusion criteria were as follows: (i) full-scale IQ <85, as estimated using the two subtest version of the Wechsler Abbreviated Scale of Intelligence (Wechsler, 1999); (ii) presence of a pervasive developmental disorder (e.g., autism) or chronic physical illness; and (iii) any contraindication to brain scanning (e.g., claustrophobia). To equate groups for IQ, HCs with IQs >115 were excluded. Of note, we obtained detailed information about lifetime ADHD symptoms from all participants using the K-SADS-PL. The study was approved by the Suffolk National Health Service Research Ethics Committee and written informed consent was obtained from all participants.

### 2.2 Magnetic resonance imaging (MRI) data acquisition

Structural MRI data were acquired using a 3-Tesla Siemens Tim Trio scanner at the Medical Research Council Cognition and Brain Sciences Unit, Cambridge, UK. We acquired T1-weighted three-dimensional (3D) magnetization-prepared rapid gradient-echo images (voxel size = 1 × 1 × 1 mm, repetition time = 2250 ms, echo time = 2.99 ms, inversion time = 900 ms, flip angle = 9°). Total scanning time was 4 min 16 s. These data were acquired at the start of the scanning session and were visually inspected for quality by the research team and an experienced radiographer. We repeated the structural MRI sequence if there was any evidence of motion artifacts in the first scan.

#### 2.2.1 SBM metrics: cortical thickness, surface area (SA) and local gyrification index (lGI)

MRI-based quantification of cortical thickness, SA and lGI was performed using FreeSurfer 5.1.0 (http://surfer.nmr.mgh.harvard.edu). This method has been described in detail in a previous study (Fischl, 2012). Briefly, the procedure involves segmentation of white matter, tessellation of the gray-white matter junction, inflation of the folded surfaces and automatic correction of topological defects in the resulting manifolds to construct representations of the gray/white matter boundary and the cortical surface. This approach uses both intensity and continuity information from the entire 3D MRI volume in segmentation and deformation procedures, and employs spatial intensity gradients across tissue classes instead of relying on absolute signal intensity. Successively, each individual's entire cortex was visually inspected and, if needed, manually edited by one of the authors (N.T.), who was blind to participant group status. This involved: (i) realignment of each subject's image to the Montreal Neurological Institute (MNI) template; (ii) setting intensity normalization control points where brain matter was erroneously skull-stripped; and (iii) adjustment of the watershed parameters of the skull strip. Cortical thickness measurements were obtained by reconstructing representations of the gray/white matter boundary and the cortical surface (approximately 160,000 vertices arranged in a triangular grid), where the distance between these two surfaces was calculated individually at each point/vertex across the cortical mantle.

Estimates of cortical SA were obtained by computing the change in area of each triangle when mapped into spherical atlas space through allocating one third of the area of each triangle to each of its vertices (Winkler et al., 2012). The lGI, which measures the degree of cortical folding within a sulcus versus that outside the sulcus, was calculated according to the method described by Schaer et al. (2008). In order to map all subjects' brains to a common space, reconstructed surfaces were registered to an average cortical surface atlas using a nonlinear procedure that optimally aligned sulcal and gyral features across subjects (Fischl et al., 1999a,b).

#### 2.2.2 Statistical analyses

In order to perform vertex-by-vertex cluster analysis, the vertex-wise cortical thickness, SA, and lGI maps for all subjects were converted to a common atlas space by applying the transformations computed in the previous step. For each hemisphere, group differences in cortical thickness at each vertex (i.e., CD > HC and vice versa) were tested using a general linear model (GLM) that included number of lifetime ADHD symptoms as a covariate (in Supplementary Tables 1–3, we report results from analyses in which number of ADHD symptoms was not included as a covariate). Given previous evidence showing that CO-CD and AO-CD may be distinguished on a quantitative basis in terms of brain structural or functional abnormalities (Fairchild et al., 2013b; Passamonti et al., 2010), we ran analyses comparing these subgroups (i.e., CO-CD > AO-CD, AO-CD > CO-CD). If there were no differences between the CD subgroups, they were treated as a combined group in the comparisons with the HC group. Furthermore, separate GLM regression analyses were carried out within the CD group alone to investigate the relationships between regional cortical thickness, SA, lGI and: (i) CU traits; and (ii) number of lifetime CD symptoms.

The level of statistical significance was evaluated using a cluster-wise P (CWP) value correction procedure for multiple comparisons based on a Monte Carlo z-field simulation (Hyatt et al., 2012). Clusters were only reported if they met a stringent whole-brain corrected threshold of CWP ≤ 0.001.

## 3 Results

### 3.1 Participants

Table 1 summarizes the demographic and clinical characteristics of the sample. As expected, CD individuals scored higher than HCs in terms of total psychopathic and CU traits, and number of CD and ADHD symptoms. Post-hoc tests comparing the CO-CD and AO-CD subgroups revealed that CO-CD youths endorsed more lifetime CD (*P* = 0.03) and ADHD symptoms (*P* = 0.04) than AO-CD participants, but they were matched on all other variables. The CO-CD and AO-CD groups did not differ significantly from HCs in age or SES, and there were no differences between the HC and AO-CD groups in IQ. However, the CO-CD group had lower estimated full-scale and verbal IQs than the HC group (both *P* < 0.05), although they were matched in terms of performance IQ (*P* = 0.34).

### 3.2 Group comparisons for cortical thickness, surface area and local gyrification index

**Fig. 1:**
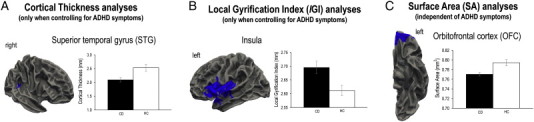
Group differences in cortical thickness, folding and surface area. A. Reduced superior temporal gyrus (STG) cortical thickness in youths with conduct disorder (CD) relative to healthy controls (HCs). Note that this finding was only significant when controlling for comorbid attention-deficit/hyperactivity disorder (ADHD) symptoms. B. Similarly, when ADHD symptoms *were* included as a covariate, youths with CD showed increased cortical folding (as assessed using local gyrification index or lGI) in the left insula relative to HCs. C. Cortical surface area (SA) in the left OFC was reduced in youths with CD relative to HCs, and this result was independent of ADHD comorbidity.

As ADHD comorbidity significantly modulated the group effects for some of these variables, for the sake of clarity we focus on the findings obtained when number of lifetime ADHD symptoms was included as a covariate. In this set of analyses, we found that youths with CD showed reduced cortical thickness in the right posterior superior temporal gyrus (STG) relative to HCs (Fig. 1A and Table 2). There were no significant differences between the CO-CD and AO-CD subgroups in cortical thickness. Participants with CD showed increased lGI in the left insula (Fig. 1B), left fusiform gyrus and right rostral middle frontal gyrus relative to HCs (Table 3). In addition, CO-CD participants showed increased lGI in the left superior frontal gyrus, left inferior temporal gyrus, right superior parietal lobule and right fusiform gyrus, relative to AO-CD participants (Table 3). Given these differences between the CD subgroups, we compared each CD subgroup with the HCs in separate analyses. Relative to HCs, CO-CD participants showed increased lGI in several frontal and temporal regions including the insula, whereas AO-CD participants displayed increased lGI in the fusiform gyrus and insula (Table 3). Finally, we found that participants with CD showed reduced surface area (SA) in the OFC compared with HCs (Fig. 1C and Table 4), and again there were no differences between the CD subgroups in SA.

The SA results obtained when number of lifetime ADHD symptoms was not included as a covariate were broadly similar to those reported above, with CD participants showing reduced SA in the OFC relative to HCs. However, the group difference in superior temporal gyrus cortical thickness was rendered non-significant, and the CD group showed increased lGI in the OFC, rather than the insula, relative to HCs (see Supplementary Tables 1–3 for further information).

### 3.3 Correlations between SBM measures and CU traits or CD symptoms in the CD group

**Fig. 2:**
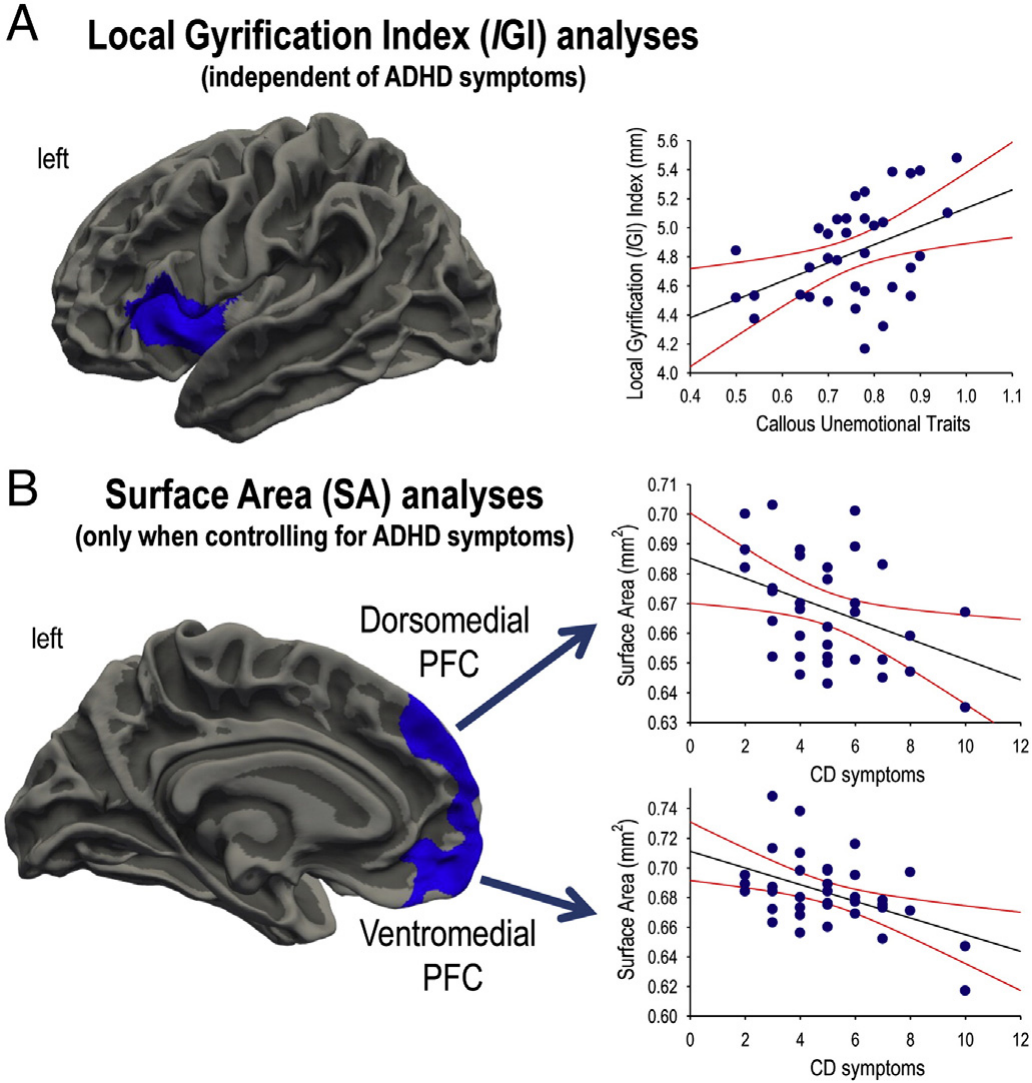
Callous–unemotional traits and conduct disorder symptoms were associated with changes in cortical folding and surface area. A. Callous–unemotional traits were positively correlated with cortical folding (as measured using local gyrification index, lGI) in the left insula in the conduct disorder (CD) group. This result was independent of attention-deficit/hyperactivity disorder (ADHD) comorbidity. B. Cortical surface area (SA) in the dorsomedial prefrontal cortex (PFC) and ventromedial PFC-orbitofrontal cortex was negatively correlated with the number of lifetime CD symptoms in the CD group. Note that these SA findings were only significant when controlling for comorbid ADHD symptoms.

There was a negative correlation between CU traits and lingual gyrus cortical thickness, whereas number of lifetime CD symptoms was negatively correlated with inferior parietal lobule cortical thickness (Table 2). Furthermore, there was a highly significant positive correlation between CU traits and anterior insula lGI (Fig. 2A and Table 3), and a negative correlation between number of lifetime CD symptoms and lGI in the precentral gyrus (Table 3). Finally, SA in the left dorsal prefrontal cortex (PFC) and ventromedial PFC/OFC was negatively correlated with number of lifetime CD symptoms (Fig. 2B and Table 4). These findings were independent of ADHD comorbidity, with the exception of the correlation between CD symptoms and PFC surface area which was only significant when controlling for ADHD symptoms (see Tables 2–4 and Supplementary Tables 1–3).

## 4 Discussion

The current study demonstrates the value of applying advanced surface-based morphometry methods to investigate brain anatomical changes in CD, as alterations in cortical thickness, surface area (SA), or folding in CD were not restricted to the cortical regions identified in previous VBM studies. Overall, this reinforces the view that cortical thickness, surface area (SA), and folding metrics each provide unique information (Hutton et al., 2009; Raznahan et al., 2011). We observed reduced cortical thickness in the superior temporal gyrus and reduced OFC SA in youths with CD relative to HCs. There was also a negative correlation between OFC SA and number of CD symptoms. Increased insula folding was observed in youths with CD relative to HCs, and this appeared to be driven by higher levels of CU traits within the CD group. However, the inclusion or exclusion of ADHD symptoms as a covariate in the statistical models significantly modulated the group effects obtained for cortical thickness and folding. Specifically, superior temporal gyrus cortical thickness and insula cortical folding abnormalities were observed in the CD group when number of ADHD symptoms was included as a covariate. In contrast, there were no group differences in cortical thickness and increased OFC folding was observed in the CD group when number of ADHD symptoms was not included as a covariate. This suggests that the presence of ADHD comorbidity influences the relationship between CD and changes in cortical structure, as assessed via SBM methods. Hence, detailed measurement of psychiatric comorbidity appears to be critical in samples of this kind.

### 4.1 Cortical thickness

Consistent with previous findings (Fahim et al., 2011; Hyatt et al., 2012; Wallace et al., 2014), we observed reduced cortical thickness in the superior temporal gyrus (STG) in CD participants relative to HCs. Importantly, this finding was only significant when ADHD symptoms were included as a covariate, which is consistent with the findings of previous studies which either deliberately excluded CD youths with comorbid ADHD (Hyatt et al., 2012), or showed that changes in superior temporal gyrus cortical thickness were present in CD individuals without comorbid ADHD (Wallace et al., 2014). The posterior STG is contiguous to the temporo-parietal junction and both regions are thought to play an important role in allocating attention to emotional stimuli, theory of mind, and moral reasoning (Blair and Mitchell, 2009). Hence, STG cortical thinning may contribute to the social cognitive difficulties observed in youths with CD (Oliver et al., 2011).

We also found that cortical thickness in the lingual gyrus was negatively correlated with CU traits, whereas inferior parietal lobule cortical thickness was negatively correlated with number of CD symptoms. These findings were independent of ADHD comorbidity. These results could explain why individuals with high levels of CU traits or severe forms of CD show deficits in facial emotion recognition (Fairchild et al., 2009a; Marsh and Blair, 2008). However, future studies assessing cortical structure and emotion recognition performance in the same individuals are needed to test this hypothesis.

### 4.2 Local gyrification index (lGI)

Contrary to the findings of Hyatt et al. (2012) and our a priori hypothesis, we found *increased* rather than *decreased* cortical folding (as assessed using lGI measures) in the insula of CD youths relative to HCs. However, this effect was only significant when controlling for comorbid ADHD symptoms. Sex differences between the two studies may underlie these divergent findings, as Hyatt et al. included males and females whereas our study was restricted to male subjects. Relevant to this point, a recent VBM study demonstrated a robust sex-by-CD diagnosis interaction in the anterior insula which was driven by *increased* insula volume in CD versus HC males and *decreased* insula volume in CD versus HC females (Fairchild et al., 2013a). Hence, sex-by-diagnosis interaction effects on insular anatomy may explain the opposite findings obtained for lGI between our study and Hyatt et al.'s (2012) study, although further SBM studies with larger, mixed-sex samples are needed to test whether the relationship between cortical structure and CD differs by sex.

We also observed strong positive correlations between insula lGI and CU traits in the CD group, which were independent of ADHD comorbidity. The insula is implicated in social cognition and empathy and has been identified as a key region in the pathophysiology of CD and psychopathy (Blair and Mitchell, 2009). Furthermore, the increased gyrification observed in this region in CD participants with high levels of CU traits may represent a neurodevelopmental abnormality that leads to empathy deficits. It should be noted that increased lGI has been reported in other neurodevelopmental disorders such as autism (Wallace et al., 2013) and schizophrenia (Palaniyappan and Liddle, 2012). Interestingly, cortical folding shows a developmental overshoot in the typically-developing brain, with lGI values increasing during the prenatal period, reaching a peak at around 1.5 years of age, and subsequently declining between infancy and adulthood (Armstrong et al., 1995). Consequently, it is possible that the increases in insula lGI observed in participants with CD reflect a failure in the typical process of cell pruning and refining of connections in infancy or childhood. Alternatively, changes in lGI in CD may result from alterations in intrinsic connectivity patterns, or individual differences in the relative growth of the supragranular and infragranular layers of the cortex (Zilles et al., 1989; Zilles et al., 2013). We note that longitudinal neuroimaging studies are needed to investigate the possible neurodevelopmental basis of these lGI differences.

Finally, we observed a negative correlation between number of CD symptoms and lGI in the precentral gyrus, a region that plays a key role in motor control. Although we did not predict this finding, this result is consistent with a previous study showing that resting state activity in the precentral gyrus was altered in highly impulsive young offenders relative to HCs (Shannon et al., 2011).

### 4.3 Cortical surface area (SA)

The present results show that OFC SA was significantly reduced in CD youths relative to HCs and this finding was influenced by CD severity, as OFC SA was negatively correlated with number of CD symptoms. Structural deficits in the OFC may underlie the difficulties in reward processing, decision-making, and emotion regulation displayed by youths with CD (Fairchild et al., 2009a,b), although only one of the earlier studies showed reduced OFC volume in youths with CD, relative to HCs (Huebner et al., 2008). The present findings suggest that some of the anatomical features contributing to OFC structure are altered in CD, but that advanced SBM methods are required to reveal these relationships.

### 4.4 Comparisons between the childhood-onset and adolescence-onset CD subtypes

In line with a previous study (Hyatt et al., 2012), we found no significant differences between the CD subtypes in terms of cortical thickness. We also found that there were no significant differences between the CO-CD and AO-CD subtypes in cortical SA. Nevertheless, several frontal and temporal regions showed greater lGI in CO-CD versus AO-CD participants, in line with Hyatt et al.'s (2012) findings. Notably, the majority of the lGI differences between CO-CD and AO-CD groups were only significant when including ADHD symptoms as a covariate. In this case, we detected significantly greater lGI in CO-CD relative to AO-CD participants, in the superior frontal gyrus, inferior temporal gyrus, superior parietal lobule, and fusiform gyrus. Conversely, Hyatt et al. (2012) reported effects in the same direction in other regions including the insula, inferior frontal gyrus, STG, OFC, frontal pole, inferior parietal cortex, and precentral gyrus. In follow-up analyses, we found that both CD subgroups showed increased lGI relative to HCs, although the location of these folding abnormalities differed according to CD subtype.

Overall, the present results provide limited evidence for differences in cortical structure between the CD subtypes, although they suggest that some lGI abnormalities may distinguish between the CO-CD and AO-CD subtypes. Interestingly, we found that both CD subgroups showed increased lGI relative to HCs. The question remains whether the behavioral differences between these CD subtypes (e.g., the more persistent and severe pattern of antisocial behavior observed in CO-CD relative to AO-CD) are underpinned by the more widespread folding abnormalities that were observed in the former group (Fairchild et al., 2013b). This important issue can only be addressed by studying the developmental trajectories of cortical folding in both CD subtypes.

### 4.5 Strengths and limitations

This study had several strengths, including the detailed characterization of the sample, the use of multiple SBM metrics rather than just cortical thickness (Fahim et al., 2011) or just cortical thickness and folding (Hyatt et al., 2012), and the care that was taken to match the groups in terms of age, IQ, SES and gender. The fact that our study was restricted to males also means that the present findings are arguably easier to interpret than those obtained with mixed-sex samples (Fahim et al., 2011; Hyatt et al., 2012; Wallace et al., 2014). This study was also only the second to directly compare the childhood-onset and adolescence-onset forms of CD using cortical thickness and folding measures, and the first one to compare these subgroups in terms of cortical surface area.

In terms of potential limitations, it should be noted that a relatively large number of statistical tests were performed. This may have increased the probability of type I errors, although the use of more stringent methods to correct for multiple comparisons than were used in previous studies should have mitigated against this issue (i.e., CWP ≤ 0.001 rather than CWP < 0.05 (Fahim et al., 2011; Hyatt et al., 2012; Wallace et al., 2014)). Second, even though this was the largest SBM study of CD to date, our sample size was moderate in comparison to studies of other disorders such as schizophrenia (Goldman et al., 2009;) or autism (Wallace et al., 2013 Ecker et al., 2013). We therefore acknowledge that additional research with larger samples is needed to replicate our findings. Finally, this study relied on cross-sectional neuroimaging data and therefore our results require extension using longitudinal designs that involve repeated assessments of brain structure from childhood into adolescence. This would reveal the developmental emergence of cortical structural markers of CD and enable us to classify CD subjects as CO-CD or AO-CD without relying on retrospective accounts of age-of-onset. Nevertheless, we attempted to address this latter issue by obtaining detailed information from participants and parents and asking both informants to consider salient life landmarks (such as the transition from primary to secondary school) to assist accurate recall.

## 5 Conclusions

We observed significant differences between youths with CD and healthy controls in superior temporal gyrus cortical thickness, orbitofrontal cortex surface area, and insular cortical folding. These results are partly in line with those reported in previous studies of non-comorbid CD, but they also add to the existing literature by demonstrating changes in cortical thickness and surface area in youths with both childhood-onset and adolescence-onset forms of CD. There were differences between these CD subgroups in cortical folding in temporal and parietal regions, although both groups showed increased cortical folding relative to HCs. Lastly, our results suggested an association between callous–unemotional traits and insula folding abnormalities.

## Figures and Tables

**Supplementary Fig. 1:**
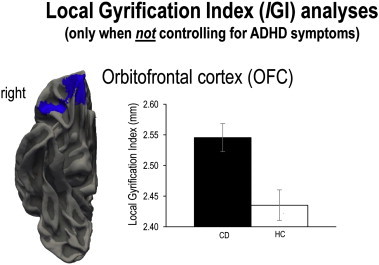
Increased cortical folding in the conduct disorder group relative to healthy controls. Cortical folding (as measured using local gyrification index or *l*GI) was increased in the right orbitofrontal cortex (OFC) of youths with CD relative to healthy controls (HCs). However, this finding was only significant when *not* controlling for comorbid ADHD symptoms.

**Table 1:** Demographic and clinical characteristics of the participants.

Measure	HC (*n* = 20)		CO-CD (*n* = 18)		AO-CD (*n* = 18)		One-way ANOVA F and P values
	Mean	SD	Mean	SD	Mean	SD	
Age (years)	18.5	1.1	18.2	0.8	18.0	0.9	*F* = 0.4; *P* = 0.7
Estimated full-scale IQ	102.4	8.1	96.4	7.8	101.6	9.6	*F* = 2.7; *P* = 0.08
Verbal IQ	49.3	6.8	44.4	6.9	47.9	7.6	*F* = 2.3; *P* = 0.1
Performance IQ	53.9	5.8	52.0	6.1	53.6	6.4	*F* = 0.5; *P* = 0.6
Psychopathic traits (YPI total)	2.0	0.3	2.6	0.4	2.5	0.3	*F* = 16.6; *P* < 0.001
CU traits (YPI CU subscale)	0.6	0.1	0.8	0.1	0.7	0.1	*F* = 10.1; *P* < 0.001
Lifetime CD symptoms	0.4	0.7	9.5	1.5	8.0	2.5	*F* = 152.5; *P* < 0.0001
Aggressive CD symptoms	0.1	0.3	3.7	1.0	3.1	1.5	*F* = 65.4; *P* < 0.0001
State anxiety (STAI)	31.2	6.5	28.1	6.3	30.7	6.2	*F* = 1.3; *P* = 0.3
Trait anxiety (STAI)	33.5	5.7	40.6	9.8	38.4	8.4	*F* = 3.9; *P* = 0.03
Lifetime ADHD symptoms	2.6	2.5	9.8	4.7	6.4	4.7	*F* = 15.4; *P* < 0.001

ACORN socioeconomic status	n	%	n	%	n	%	χ^2^ (exact)

Wealthy achievers	3	15	0	0.0	1	2.8	*P* = 0.32
Urban prosperity	5	25	2	5.6	4	11.1	
Comfortably off	4	20	6	16.7	5	13.9	
Moderate means	2	10	2	5.5	0	0.0	
Hard-pressed	6	30	8	22.2	8	22.2	

Key to abbreviations: ADHD, Attention-Deficit/Hyperactivity Disorder; AO-CD, adolescence-onset Conduct Disorder; CO-CD, childhood-onset Conduct Disorder; CU, callous–unemotional; HC, healthy control; IQ, intelligence quotient; SD, standard deviation; STAI, State-Trait Anxiety Inventory; YPI, Youth Psychopathic traits Inventory. Note: ACORN is a geodemographic tool for assessing socioeconomic status using postcodes.

**Table 2:** Summary of the cortical thickness results obtained when including number of lifetime attention-deficit/hyperactivity disorder symptoms as a covariate.

	Brain region	Hemisphere	NVtxs	Size (mm^2^)	X	Y	Z	Max	CWP
*Group comparisons*									
HC > CD	Superior temporal gyrus	R	338	154.4	64	−37	13	3.5	0.001
CD > HC	None significant at CWP ≤ 0.001								
CO-CD > AO-CD and vice-versa	None at CWP ≤ 0.001								

*Correlation with CU traits in the CD group*									
Negative correlation	Lingual gyrus	L	399	168.9	−22	−54	−2	4.3	0.0007
Positive correlation	None at CWP ≤ 0.001								

*Correlation with lifetime CD symptoms in the CD group*									
Negative correlation	Inferior parietal lobule	R	344	184.6	45	−64	32	3.5	0.0002
Positive correlation	Inferior frontal gyrus (pars opercularis)	R	297	169.5	47	25	20	3.2	0.0007

Key to abbreviations: AO-CD, adolescence-onset CD; CD, conduct disorder; CO-CD, childhood-onset CD; CU, callous–unemotional; CWP, cluster-wise-*P* value; HC, healthy control; NVtxs, number of vertices; Max, maximum −log10(*P* value) in the cluster.

**Table 3:** Summary of the local gyrification index (lGI) results obtained when including number of lifetime attention-deficit/hyperactivity disorder symptoms as a covariate.

	Brain region	Hemisphere	NVtxs	Size (mm^2^)	X	Y	Z	Max	CWP
*Group comparisons*									
CD > HC	Fusiform Gyrus	L	5888	2769.4	−31	−37	−23	3.9	0.0001
	Insula	L	12,785	5420.4	−34	7	−13	3	0.0001
	Rostral middle frontal gyrus	R	1930	1300.8	40	48	3	4.1	0.0001
HC > CD	None at CWP ≤ 0.001								
CO-CD > AO-CD	Superior frontal gyrus	L	4396	1847.7	−18	7	64	2.6	0.0001
	Inferior temporal gyrus	L	2221	1138.8	−42	−13	−27	1.8	0.0005
	Superior parietal lobule	R	4413	1960.9	18	−66	52	3	0.0001
	Fusiform gyrus	R	1967	1252.6	35	−64	−16	2.9	0.0001
CO-CD > HC	Inferior temporal gyrus	L	7128	3528.4	−41	−14	−26	4.3	0.0001
	Superior frontal gyrus	L	3572	1811.3	−19	12	53	3.4	0.0001
	Insula	L	11,966	5026.9	−34	7	−13	2.9	0.0001
	Rostral middle frontal gyrus	R	2788	1854.6	40	50	5	3.7	0.0001
	Paracentral gyrus	R	5254	1982.1	6	−32	53	3.1	0.0001
AO-CD > HC	Fusiform gyrus	L	4204	1896.8	−31	−37	−23	3.1	0.0001
	Insula	L	5238	2164.6	−26	17	−14	2.7	0.0001
AO-CD > CO-CD	None at CWP ≤ 0.001								
HC > CO-CD and HC > AO-CD	None at CWP ≤ 0.001								

*Correlation with CU traits in the CD group*									
Negative correlation	None at CWP ≤ 0.001								
Positive correlation	Insula	L	6749	2771.7	−33	18	−4	3.2	0.0001

*Correlation with lifetime CD symptoms in the CD group*									
Negative correlations	Lateral occipital cortex	L	3268	2220.9	−25	−92	7	4	0.0001
	Precentral gyrus	L	4195	1737.9	−19	−28	55	3	0.0001
Positive correlation	Precuneus	R	8281	3203	8	−42	39	2.8	0.0001

Key to abbreviations: AO-CD, adolescence-onset CD; CD, Conduct Disorder; CO-CD, childhood-onset CD; CU, callous–unemotional; CWP, cluster-wise-*P* value; HC, healthy control; NVtxs, number of vertices; Max, maximum −log10(*P* value) in the cluster.

**Table 4:** Summary of the cortical surface area (SA) results obtained when including number of lifetime attention-deficit/hyperactivity disorder symptoms as a covariate.

	Brain region	Hemisphere	NVtxs	Size (mm^2^)	X	Y	Z	Max	CWP
*Group comparison*									
HC > CD	Orbitofrontal cortex	L	1975	1466.2	−7	52	−15	4.1	0.0005
CD > HC	None at CWP ≤ 0.001								
CO-CD > AO-CD and vice-versa	None at CWP ≤ 0.001								

*Correlation with lifetime CD symptoms in the CD group*									
Negative correlation	Ventromedial PFC extending to dorsomedial PFC	L	2285	1451.0	−9	48	−12	2.7	0.0007

Key to abbreviations: AO-CD, adolescence-onset CD; CD, conduct disorder; CO-CD, childhood-onset CD; CWP, cluster-wise-*P* value; HC, healthy control; NVtxs, number of vertices; Max, maximum −log10(*P* value) in the cluster; PFC, prefrontal cortex.

## Appendix A Supplementary data

Supplementary Table 1. Summary of the cortical thickness results obtained when the number of lifetime attention-deficit/hyperactivity disorder (ADHD) symptoms was not included as a covariate. Supplementary Table 2. Summary of the local gyrification index (lGI) results obtained when the number of lifetime attention-deficit/hyperactivity disorder (ADHD) symptoms was not included as a covariate. Supplementary Table 3. Summary of the cortical surface area (SA) results obtained when the number of lifetime attention-deficit/hyperactivity disorder (ADHD) symptoms was not included as a covariate.
