# Supplementary material for: Cortical thickness, surface area, and folding alterations in male youths with conduct disorder and varying levels of callous–unemotional traits
Source: Neuroimage Clin. 2015 Apr 30;8:253–60. doi: 10.1016/j.nicl.2015.04.018 (PMC4473851; doi:10.1016/j.nicl.2015.04.018)
Supplement: Supplementary file 1 — Supplementary Table 1. Summary of the cortical thickness results obtained when the number of lifetime attention-deficit/hyperactivity disorder (ADHD) symptoms was not included as a covariate. Supplementary Table 2. Summary of the local gyrification index (lGI) results obtained when the number of lifetime attention-deficit/hyperactivity disorder (ADHD) symptoms was not included as a covariate. Supplementary Table 3. Summary of the cortical surface area (SA) results obtained when the number of lifetime attention-deficit/hyperactivity disorder (ADHD) symptoms was not included as a covariate. [file mmc1.doc]

**Supplementary Table 1.** Summary of the cortical thickness results obtained when the number of lifetime Attention-Deficit/Hyperactivity Disorder (ADHD) symptoms was *not* included as a covariate.

| **Group comparisons** | **Brain Region** | **Hemisphere** | **NVtxs** | **Size (mm^2)** | **X** | **Y** | **Z** | **Max** | **CWP** |
| --- | --- | --- | --- | --- | --- | --- | --- | --- | --- |
| CD > HC | None at CWP≤0.001 |  | | | | | | | |
| HC > CD | None at CWP≤0.001 |  | | | | | | | |
| AO-CD > CO-CD and *vice versa* | None at CWP≤0.001 |  | | | | | | | |
| **Correlation with CU traits in the CD group** | **Brain Region** | **Hemisphere** | **NVtxs** | **Size (mm^2)** | **X** | **Y** | **Z** | **Max** | **CWP** |
| Negative correlation | Lingual Gyrus | L | 453 | 190.1 | -22 | -54 | -2 | 4.0 | 0.0002 |
| Positive correlation | None at CWP≤0.001 |  | | | | | | | |
| **Correlation with lifetime CD symptoms in CD group** | **Brain Region** | **Hemisphere** | **NVtxs** | **Size (mm^2)** | **X** | **Y** | **Z** | **Max** | **CWP** |
| Negative correlations | Inferior Parietal Lobule | R | 333 | 178.4 | 40 | -69 | 40 | 3.9 | 0.0003 |
|  | Inferior Parietal Lobule | L | 262 | 165.2 | -31 | -68 | 42 | 4.0 | 0.0009 |
| Positive correlation | Rostral Middle Frontal Gyrus | R | 295 | 167.4 | 45 | 27 | 26 | 3.1 | 0.0007 |

**Key to abbreviations:** AO-CD, adolescence-onset CD; CD, Conduct Disorder; CO-CD, childhood-onset CD; CU, callous-unemotional; CWP, cluster-wise-P value; HC, healthy control; NVtxs, number of vertices; Max, maximum -log10(p value) in the cluster.

**Supplementary Table 2.** Summary of the local gyrification index (*l*GI) results obtained when the number of lifetime Attention-Deficit/Hyperactivity Disorder (ADHD) symptoms was *not* included as a covariate.

| **Group comparison** | **Brain Region** | **Hemisphere** | **NVtxs** | **Size(mm^2)** | | **X** | **Y** | **Z** | **Max** | **CWP** |
| --- | --- | --- | --- | --- | --- | --- | --- | --- | --- | --- |
| CD > HC | Parahippocampal Cortex | L | 3848 | 1703.3 | | -17 | -38 | -8 | 3.0 | 0.0001 |
|  | Orbitofrontal Cortex | R | 2574 | 1521 | | 42 | 27 | -14 | 3.5 | 0.0001 |
| HC > CD | Superior Parietal Lobule | L | 1955 | 1468.5 | | -10 | -93 | 25 | 2.4 | 0.0002 |
| CO-CD > AO-CD | Lateral Occipital Cortex | R | 1711 | 1116.9 | | 40 | -76 | -14 | 2.4 | 0.0009 |
| AO-CD > CO-CD | None at CWP≤0.001 |  | | | | | | | | |
| **Correlation with CU traits in the CD group** | **Brain region** | **Hemisphere** | **NVtxs** | | **Size(mm^2)** | **X** | **Y** | **Z** | **Max** | **CWP** |
| Negative correlation | None at CWP≤0.001 |  | | | | | | | | |
| Positive correlation | Insula | L | 5985 | | 2387.6 | -37 | 0 | -20 | 3.3 | 0.0001 |
| **Correlation with lifetime CD symptoms in CD group** | **Brain region** | **Hemisphere** | **NVtxs** | | **Size(mm^2)** | **X** | **Y** | **Z** | **Max** | **CWP** |
| Negative correlations | Lateral Occipital Cortex | L | 3898 | | 2558.2 | -23 | -93 | 6 | 4 | 0.0001 |
|  | Precentral Gyrus | L | 4032 | | 1672.2 | -22 | -26 | 53 | 2.6 | 0.0001 |
|  | Rostral Middle Frontal Gyrus | R | 2006 | | 1380.2 | 39 | 45 | 2 | 4.2 | 0.0001 |
| Positive correlations | Cuneus | L | 1804 | | 1248.8 | -5 | -79 | 12 | 2.1 | 0.0003 |
|  | Precuneus | R | 5234 | | 1966.5 | 8 | -42 | 39 | 2.6 | 0.0001 |

**Key to abbreviations:** AO-CD, adolescence-onset CD; CD, Conduct Disorder; CO-CD, childhood-onset CD; CU, callous-unemotional; CWP, cluster-wise-P value; HC, healthy control; NVtxs, number of vertices; Max, maximum -log10(p value) in the cluster; YPI, Youth Psychopathic traits Inventory.

**Supplementary Table 3.** Summary of the cortical surface area (SA) results obtained when the number of lifetime Attention-Deficit/Hyperactivity Disorder (ADHD) symptoms was *not* included as a covariate.

| **Group comparison** | **Brain Region** | **Hemisphere** | **NVtxs** | **Size(mm^2)** | **X** | **Y** | **Z** | **Max** | **CWP** |
| --- | --- | --- | --- | --- | --- | --- | --- | --- | --- |
| HC > CD | Orbitofrontal cortex | L | 1741 | 1322.2 | -7 | 52 | -15 | 4.2 | 0.001 |
| CD > HC | None at CWP≤0.001 |  | | | | | | | |
| AO-CD > CO-CD and vice versa | None at CWP≤0.001 |  | | | | | | | |

**Key to abbreviations:** AO-CD, adolescence-onset CD; CD, Conduct Disorder; CO-CD, childhood-onset CD; CWP, cluster-wise-P value; HC, healthy control; NVtxs, number of vertices; Max, maximum -log10(p value) in the cluster.
